# Supplementary material for: HIVprotI: an integrated web based platform for prediction and design of HIV proteins inhibitors
Source: J Cheminform. 2018 Mar 9;10:12. doi: 10.1186/s13321-018-0266-y (PMC5845081; doi:10.1186/s13321-018-0266-y)
Supplement: Supplementary file 2 — Additional file 2. Source code of HIVProtI web server. [file 13321_2018_266_MOESM2_ESM.zip › HIVprotI_Source-code/hivprotI/weka-3-6-6/doc/weka/classifiers/functions/supportVector/package-summary.html]

weka.classifiers.functions.supportVector


---


|  |  |  |
| --- | --- | --- |
| **Overview** \| **Package** \| Class \| **Tree** \| **Deprecated** \| **Index** \| **Help** \| **Weka's home** | |  |
| **PREV PACKAGE**   **NEXT PACKAGE** | **FRAMES**    **NO FRAMES**     **All Classes** |  |


---

## Package weka.classifiers.functions.supportVector

| **Class Summary** | |
| --- | --- |
| **CachedKernel** | Base class for RBFKernel and PolyKernel that implements a simple LRU. |
| **CheckKernel** | Class for examining the capabilities and finding problems with kernels. |
| **Kernel** | Abstract kernel. |
| **KernelEvaluation** | Class for evaluating Kernels. |
| **NormalizedPolyKernel** | The normalized polynomial kernel.  K(x,y) = <x,y>/sqrt(<x,x><y,y>) where <x,y> = PolyKernel(x,y)   Valid options are: |
| **PolyKernel** | The polynomial kernel : K(x, y) = <x, y>^p or K(x, y) = (<x, y>+1)^p   Valid options are: |
| **PrecomputedKernelMatrixKernel** | This kernel is based on a static kernel matrix that is read from a file. |
| **Puk** | The Pearson VII function-based universal kernel.    For more information see:    B. |
| **RBFKernel** | The RBF kernel. |
| **RegOptimizer** | Base class implementation for learning algorithm of SMOreg Valid options are: |
| **RegSMO** | Implementation of SMO for support vector regression as described in :    A.J. |
| **RegSMOImproved** | Learn SVM for regression using SMO with Shevade, Keerthi, et al. |
| **SMOset** | Stores a set of integer of a given size. |
| **StringKernel** | Implementation of the subsequence kernel (SSK) as described in [1] and of the subsequence kernel with lambda pruning (SSK-LP) as described in [2].    For more information, see    Huma Lodhi, Craig Saunders, John Shawe-Taylor, Nello Cristianini, Christopher J. |

---


|  |  |  |
| --- | --- | --- |
| **Overview** \| **Package** \| Class \| **Tree** \| **Deprecated** \| **Index** \| **Help** \| **Weka's home** | |  |
| **PREV PACKAGE**   **NEXT PACKAGE** | **FRAMES**    **NO FRAMES**     **All Classes** |  |


---
